# Dehydrozingerone promotes healing of diabetic foot ulcers: a molecular insight

**DOI:** 10.1007/s12079-022-00703-0

**Published:** 2022-10-25

**Authors:** Farmiza Begum, Suman Manandhar, Gautam Kumar, Raghuvir Keni, Runali Sankhe, Prasada Chowdari Gurram, Fathima Beegum, Meka Sai Teja, Krishnadas Nandakumar, Rekha R. Shenoy

**Affiliations:** Department of Pharmacology, Manipal College of Pharmaceutical Sciences, Manipal Academy of Higher Education, Manipal, Karnataka 576104 India

**Keywords:** Dehydrozingerone, Diabetic foot ulcers, High fat diet, Cellular mechanism, Inflammation

## Abstract

**Introduction:**

One of the most common problems of diabetes are diabetic foot ulcers (DFUs). According to National Institute for Health, initial management of DFUs can decrease the complication of limb amputations and can improve the patient’s quality of life. DFU treatment can be optimized with the help of multidisciplinary approach. Based on many studies, control of glucose levels in blood, antioxidant activity, reduction in cytokine levels, re-epithelialization, collagen formation, migration of fibroblasts are major phases involved in managing DFU. Dehydrozingerone (DHZ), has been known for its anti-inflammatory, antioxidant and wound healing properties.

**Methodology:**

Three months high-fat diet and low dose of streptozotocin-induced type-II diabetic foot ulcer model was used to evaluate the effectiveness of dehydrozingerone. DHZ was given orally to rats for 15 days post wounding. TNF-α, IL-1β and antioxidant parameters like lipid peroxidation, glutathione reductase were estimated. Immunoblotting was done to investigate the effect of DHZ on the expression of ERK, JNK, HSP-27, P38, SIRT-1, NFκB, SMA, VEGF and MMP-9 in skin tissue. Histopathology was performed for analyzing DHZ effect on migration of fibroblasts, formation of epithelium, granulation tissue formation, angiogenesis and collagen formation.

**Results:**

DHZ decreased the levels of malondialdehyde, TNF-α, IL-1β and increased glutathione levels in wound tissue. Western blotting results suggested that DHZ activated ERK1/2/JNK/p38 signaling, increased expression of HSP-27, SIRT-1, VEGF, SMA thus facilitating the migration and proliferation of fibroblasts, angiogenesis and decreased inflammation. Masson Trichrome & histopathology showed an increase in collagen, epithelial and granulation tissue formation.

**Conclusion:**

DHZ significantly accelerates the healing of diabetic foot ulcers in high fat diet fed plus low dose streptozotocin induced type-II diabetic Wistar rats.

**Graphical abstract:**

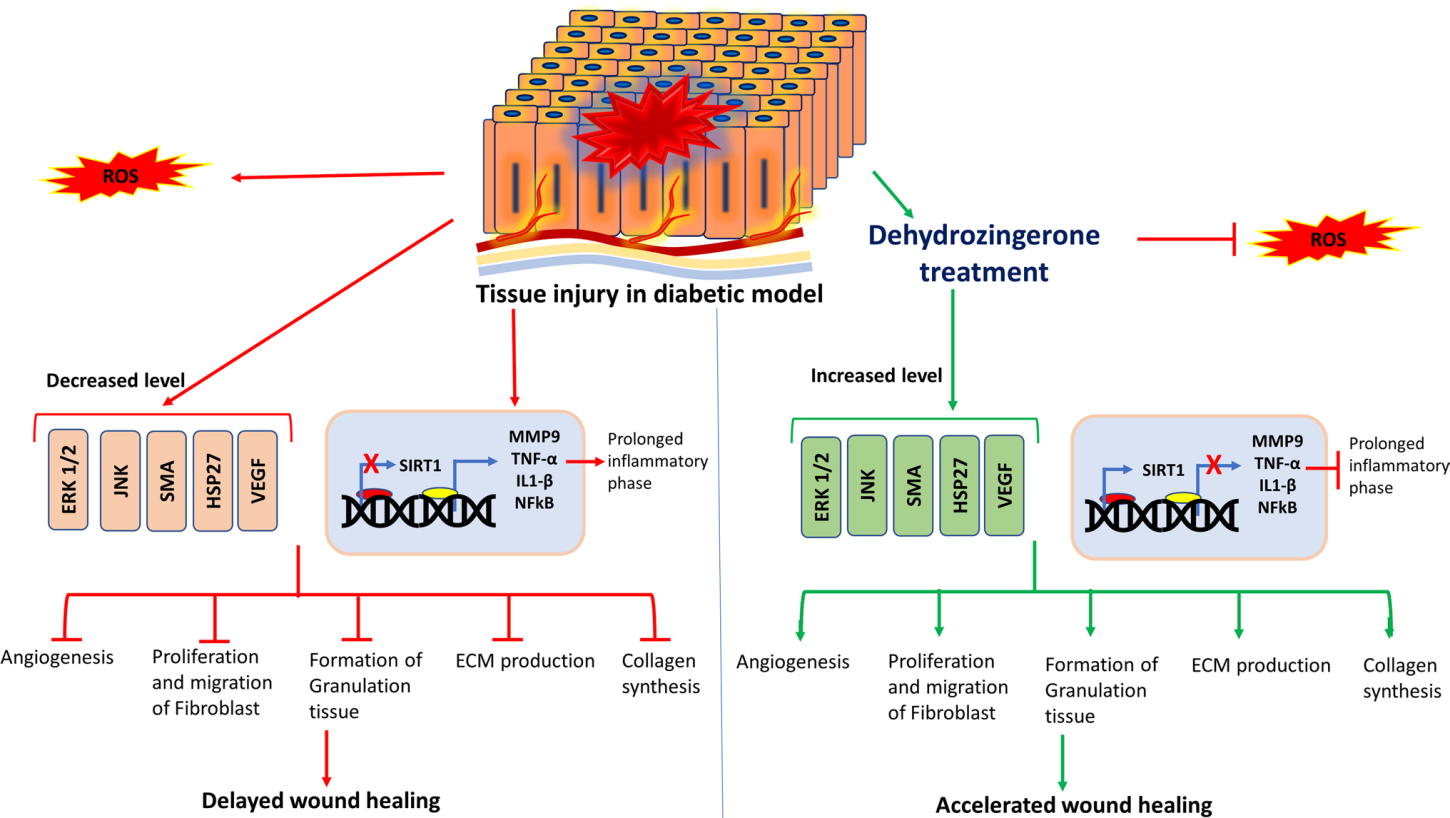

## Introduction

Diabetes mellitus is a metabolic disorder that is related to many complications and involves abnormal secretion of insulin and function of pancreatic beta cells. According to the International Diabetic Federation in 2017, about 425 million adults were diabetic and it would enhance to 629 million by 2045 (Tripathy 2018). Increasing incidence of diabetes is of growing concern. Among the adults with diabetes, 79% are living in developing countries. Diabetes is associated with numerous complications such as cardiovascular, diabetic nephropathy, neuropathy and diabetic foot ulcer (DFU). DFU is a prime difficulty in diabetes and if untreated can lead to lower limb amputations. The main cause of more than half of non-traumatic leg amputations is diabetic foot ulcers. The prevalence of DFU is suggested to be 1.3–12% in different studies and diabetic lower limb amputation is 1.5–7% (Khalifa 2018). The reason behind DFU is neuropathy, peripheral artery disease, biochemical problems and impaired wound healing. Besides lower limb amputation, DFU can lead to infection and even death. Socioeconomic implications are also associated with DFU. Studies reported that hospital cost for managing complicated DFU was estimated to be about 188,000 US dollars (Del Core et al. 2018).

Diabetes delays the wound healing process by impairing the phases of wound recovery viz., haemostasis, inflammation, proliferation and remodelling. Main features of diabetic wound restoration encompass persistent anti-inflammatory phase, impaired granulation tissue formation, angiogenesis and reduction in wound tensile strength. There are exclusive pathways responsible for delayed wound healing in diabetes (Barrientos et al. 2008; JF et al. 2011). Unlike normal wound recovery, in diabetic wound healing the number of macrophages in the wound site is greater which ends up in a persistent inflammatory phase. There is reduced growth factor release and elevated interleukins. Reduction in growth factor release, results in reduced fibroblast proliferation, migration and increased apoptosis of the cells. Reduction in vascular endothelial growth factor (VEGF) impairs activation of endothelial nitric oxide synthetase, prevents endothelial progenitor cell immobilization and impairs angiogenesis. Increased levels of matrix metalloproteases (MMP) and decreased levels of tissue inhibitor of matrix metalloproteases impair extracellular matrix (ECM) improvement. Differentiation of fibroblast to myofibroblast is reduced. Decreased migration and proliferation of keratinocytes and decreased chemokines impair angiogenesis thereby delaying wound recovery (Patel et al. 2019).

The treatment strategy for the management of DFU is to introduce a multidisciplinary approach. Management involves the right classification of stage and severity, control of diabetes mellitus, infection and improvement of blood flow. DFU treatment focuses on better perfusion, pressure mitigation and infection control. Advanced technology has resulted in a series of therapeutics like skin substitutes, tissues from bioengineering, hyperbaric oxygen, negative pressure wound therapy, wound dressings with growth factors and nanotechnology (Kasiewicz and Whitehead 2017; Perez-Favila et al. 2019). Of the above-mentioned therapies, a common feature among them is that their cost is exorbitant, they have been investigated for years and are still inaccessible to people. Use of skin substitutes involves risk of rejection (although derma graft uses bioengineered & synthetic options wherein cells and scaffolds are used still they are years away from application) (Keni et al. 2022; Nicholas and Yeung 2017). Usage of growth factors involves the risk of malignancy which is reported in various cases (Hart et al. 2012; Keni et al. 2022). Natural products obtained from plants that can also be synthesized chemically like their isolates, ayurvedic herbals and extracts are not broadly researched areas for managing foot ulcers. Many plant products have been chemically synthesized like Curcumin (Merrell et al. 2009), Sesamol (Gourishetti et al. 2020), Naringin (Kandhare et al. 2014), Resveratrol (Huang et al. 2019)  and *Syzygium cumini* (Singla et al. 2017) which have been tested and have known to accelerate healing of foot ulcers by various mechanisms involved..

It would be worthwhile screening thousands of plant products that would aid wound repair. (Keni et al. 2022). One such plant-based product is Dehydrozingerone (DHZ), which is a natural antioxidant and present in the rhizomes of *Zingiber officinale* (Ginger), resembles the half structure of curcumin (Yogosawa et al. 2012). Chemically it is 4-(4-hydroxy-3-methoxyphenyl)-3buten—one (Hayun et al. 2018). It exhibits anti-inflammatory, antimicrobial and cytotoxic activity along with its antioxidant activity (Rao et al. 2011)**.** Wound healing effect of DHZ was reported in normal wounds (Rao et al. 2011) but its activity in diabetic foot ulcers is not yet established. The results from the previous articles suggest that it might be a promising molecule for developing suitable formulations that can heal diabetic foot ulcers.

## Materials and methods

Streptozotocin procured from MP Biomedicals India Pvt. Ltd. (Navi Mumbai, India), Pierce BCA Protein Estimation Kit from Thermo Fisher Scientific India Pvt Ltd. (Mumbai, India), Primary Antibodies (ERK, p-ERK, SMA, SIRT-1, JNK, p-JNK, VEGF, MMP-9, P38, p-P38) and Secondary Antibody (HRP Labelled) from ELabscience Inc. (Wuhan, China), Westar Antares Chemiluminescent Substrate (Cyanagen, Bologna, Italy), GS-PVDF-304 membrane obtained from iScience Innovation, TNF-alpha and IL-1β ELISA kits procured from Krishgen Biosystems (Worli, Mumbai), Dehydrozingerone was synthesized in the laboratory, Contour Plus glucometer and glucose strips were procured from Ascensia Diabetes Products (Bangalore).

### Animals

Four-week-old male Wistar rats were used from Central Animal Research Facility (CARF) MAHE, Manipal and Committee for Purpose of Control & Supervision of Experiments on Animals (CPCSEA) guidelines was followed for their experimentation on animals and maintenance of food, water and light. Institutional Animal Ethics Committee (IAEC), Manipal Academy of Higher Education, Manipal approved the animal studies (IAEC/KMC/18/2019).

### Induction of type II diabetes

Animals were divided into normal control, disease control and test groups. Normal control animals were fed with normal pellets, other groups fed with high-fat diet for three months to develop insulin resistance (58% fat, 25% protein, 17% carbohydrates) (Srinivasan et al. 2005). After three months of high fat diet, disease and test group animals were given a low dose of streptozotocin (STZ) (35 mg/kg) via intraperitoneal route. High fat diet is given to attain insulin resistance and STZ to induce beta cell destruction. This model develops a suitable type-2 diabetic rat model and on the other hand closely mimics the natural history of the disease events i.e., from insulin resistance to beta cell dysfunction along with the metabolic characteristics of human type 2 diabetes. (Keni et al. 2020; Srinivasan et al. 2005) Rats were kept for stabilization for four weeks to develop diabetes. Body weight was measured weekly and blood glucose levels were recorded four weeks after STZ injection.

### Oral glucose tolerance test

After three months of high-fat diet, animals were checked for insulin resistance using oral glucose tolerance test (OGTT). Animals were kept on fasting overnight followed by oral dose of glucose (2 gm/kg). Blood glucose levels were determined at 0, 15, 30, 60, 90 and 120 min of glucose oral dosing (Gourishetti et al. 2020).

### Measurement of lipid profile

Blood (300ul) was collected via retro-orbital puncture under light ether anaesthesia. Plasma separated after centrifugation of blood samples at 6000 rpm for 10 min and was used for determining the triglyceride levels, total cholesterol and HDL levels using Aspen Kits. Absorbance was measured at 540 nm using an Elx-800 plate reader.

### Creation of diabetic foot ulcer

A full-thickness foot ulcer using a six mm biopsy punch was made on the dorsal side of the hind foot of rat under anaesthesia (10 mg/kg of xylazine & 60 mg/kg of ketamine) (Gourishetti et al. 2020; Shi et al. 2018).

Once the foot ulcer was done, animals were treated using DHZ 100 mg/kg p.o., (dose was selected based on previous studies) and normal and disease control animals were given normal saline. After 15 days of treatment, animals were sacrificed and foot ulcers were collected and stored in − 80°C for further analysis. Samples collected were stored in 10% formalin for histopathology and Masson trichrome staining.

### Measurement of wound closure

On 5th, 10th and 15th days of treatment, the wounds were photographed and the wound size was measured using Image J software. The percentage of wound closure was determined using the formula:

$${\text{Percentage}}\;{\text{Closure}}\;{\text{of}}\;{\text{wound }} = \frac{{{\text{Area}}\;{\text{of}}\;{\text{wound}}\;{\text{on}}\;{\text{day}}\;0{-}{\text{Area}}\;{\text{of}}\;{\text{wound}}\;{\text{on}}\;{\text{day}}\;{5}/{1}0/{15}^{{{\text{th}}}} }}{{{\text{Area}}\;{\text{of}}\;{\text{wound}}\;{\text{on}}\;{\text{day}}\;0}} \times {1}00$$

### Antioxidant parameters

The antioxidant parameters like Lipid peroxidation (LPO) (Bilgen et al. 2019; Botsoglou et al. 1994)**,** Glutathione reductase (GSH) (Rai et al. 2019; Smith et al. 1988), total protein (Ellman et al. 1961) were assessed in the wound tissue using UV spectrophotometric and colorimetric methods.

### Immunoblotting

Homogenization of foot ulcer **s**amples using POLYTRON-800 homogenizer was done, using RIPA buffer (Radio Immuno Precipitation Assay) for lysis of cells with protease inhibitor and phosphatase inhibitor. The obtained lysate was centrifuged at 16,000 rpm for 20 min, the supernatant was collected and protein levels were estimated. 50 ug protein was separated using SDS-PAGE (10%) electrophoresis, then transferred onto PVDF (Polyvinylidene difluoride membrane) membrane. The membrane was blocked using 3% BSA in 1X TBST for 2 h. The membrane was washed three times, 10 min each using TBST then incubated with the primary antibodies at 4 °C overnight, followed by incubation with horseradish peroxidase-conjugated anti-IgG secondary antibodies for two hours. The blots were detected using ECL solution (Westar Antares, Cyanagen, Bologna, Italy) in Syngene GBox Chemi XRQ gel documentation system. Quantification of protein band intensity was done using ImageJ software and relative density was calculated in comparison to alpha-tubulin expression.

### TNF-α & IL-1B estimation

Using the foot ulcer tissue lysates, TNF-α, IL-1β was quantified using TNF-α, IL-1β Krishgen rat ELISA kits and their levels expressed as pg/mg of protein.

### Histopathology & Masson trichrome staining

Foot ulcer samples were stored in 10% formalin. Later hematoxylin & eosin and Masson trichome staining were done for granulation tissue formation, fibroblast proliferation, epidermal regeneration, angiogenesis and collagen tissue deposition.

### Statistical analysis

Results were analyzed and expressed as Mean ± SEM. Graph pad prism 8.4.2 was used for statistical analysis using one-way ANOVA followed by Tukey’s post hoc test.

## Results & discussion

### Body weight & oral glucose tolerance test (OGTT)

**Fig. 1:**
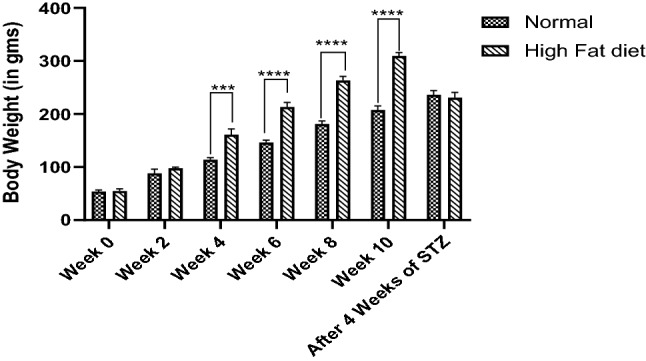
HFD effect on body weight (in gms), represented as Mean ± SEM. ****p < 0.0001 when compared to normal pellet fed diet group. Analysis done by using two-way ANOVA with Sidak’s post-hoc test

High-fat diet fed Wistar rats for three months led to weight gain (309.3 ± 6.64) when compared to Wistar rats fed with a normal pellet diet (236 ± 8.08). Weight gain continued till they gained insulin resistance i.e., till three months of HFD feeding. A reduction in weight was observed after STZ injection (230.66 ± 10.26) (Fig. 1). Weight gain in HFD-fed rats could be due to more consumption of a diet that is high in energy and fat (lard) and may be due to its deposition in various body parts when compared to normal diet-fed rats (Reed et al. 2000; Srinivasan et al. 2005).

**Fig. 2:**
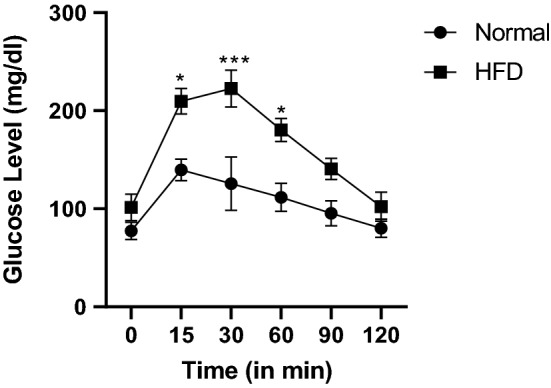
Effect of HFD on OGTT (mg/dl). Data represented as Mean ± SEM. *p < 0.01, ***p < 0.0001 when compared to normal pellet fed diet group. Analysis done by using two-way ANOVA with Sidak’s post-hoc test

After three months of HFD feeding, insulin resistance was developed which was confirmed by OGTT (Fig. 2). OGTT involved monitoring of blood glucose levels at regular time intervals for two hours. An increase in blood glucose levels was observed when compared to normal control animals which indicated impaired clearance of increased glucose from the blood. Compared to the normal group, high fat diet-fed group showed an increase in blood glucose levels at 15, 30, 60, 90, and 120 min. Once insulin resistance was obtained which is one of the reasons for type-II diabetes, STZ (35 mg/kg) was injected to animals to develop hyperglycaemia which was confirmed by measuring the blood glucose levels for four weeks. Rats with blood glucose levels between 300 and 400 mg/dl were considered for the study and divided into disease control and treatment groups. Treatment was continued for 15 days (Gourishetti et al. 2020; Kunisaki et al. 1995; Reed et al. 2000).

### Lipid profile

**Fig. 3:**
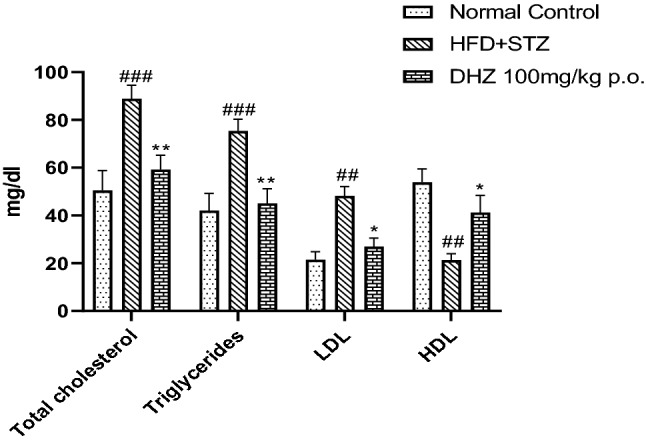
Effect of HFD on Lipid profile (mg/dl). Data represented as Mean ± SEM. **p < 0.001, *p < 0.01 when compared to HFD group and ^##^*p* < 0.001, ^###^*p* < 0.0001 compared to normal pellet fed diet. Analysis done by using two-way ANOVA with Tukey’s post-hoc test

Due to the consumption of high-fat diet, the animals became pre-diabetic attaining insulin resistance by gaining significant body weight which was confirmed by OGTT. Thus, HFD- fed rats showed increased levels of triglycerides (TG), total cholesterol (TC), LDL and decreased levels of HDL when compared to the normal diet-fed rats. Treatment with DHZ significantly restored the levels of triglycerides, total cholesterol, LDL and HDL (Fig. 3).

### Percentage wound contraction

**Fig. 4:**
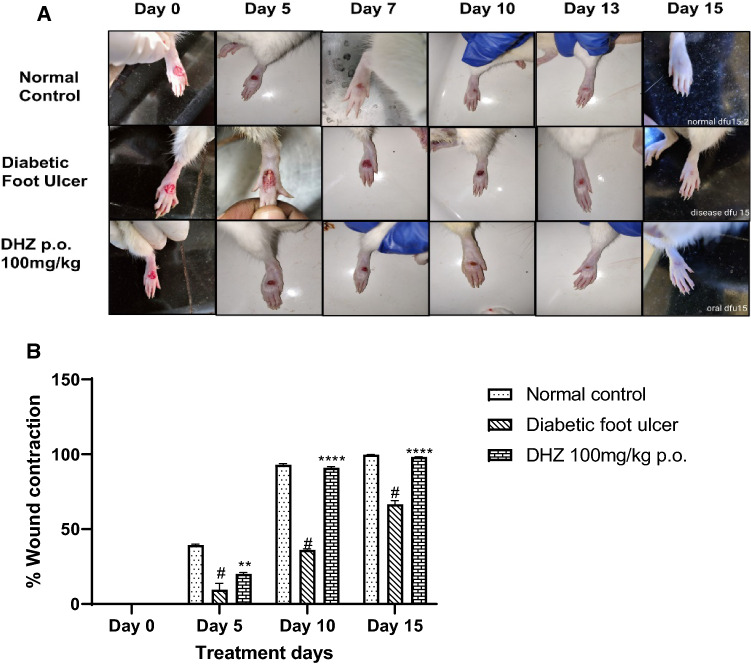
**A** Representative images of effect of DHZ treatment on different days of DFU **B** Effect of DHZ on re-epithelization of wound. Data represented as Mean ± SEM. **p < 0.001, ****p < 0.0001 when compared to HFD group and ^#^*p* < 0.001 compared to normal pellet fed diet. Analysis done by using two-way ANOVA with Tukey’s post-hoc test.

The effect of DHZ on wound healing was evaluated in a foot ulcer model in Wistar rats. Rats were treated for 15 days with DHZ 100 mg/kg p.o., and the wound area was measured on days 5, 10 and 15 respectively. Day 5th post wounding, in DHZ treated animals (20 ± 0.94) acceleration of healing was observed when compared to disease group animals (39.2 ± 0.83) (*p* < 0.001). On Day 10, DHZ showed 90% (90.23 ± 0.68) of re-epithelization when compared with normal control (92.91 ± 0.78) and disease control animals (36.02 ± 0.79) which was statistically significant with the disease control (*p* < 0.0001). At the end of day 15, re-epithelization was almost 100% in normal control and 98% in DHZ-treated animals (98.16 ± 0.10) which was statistically significant (*p* < 0.0001) compared to 66% in disease group animals (66.51 ± 2.55) (Fig. 4)**.** These results indicate that treatment with DHZ significantly hastened the wound healing when compared to the diseased group of animals.

### Antioxidant parameters

**Fig. 5:**
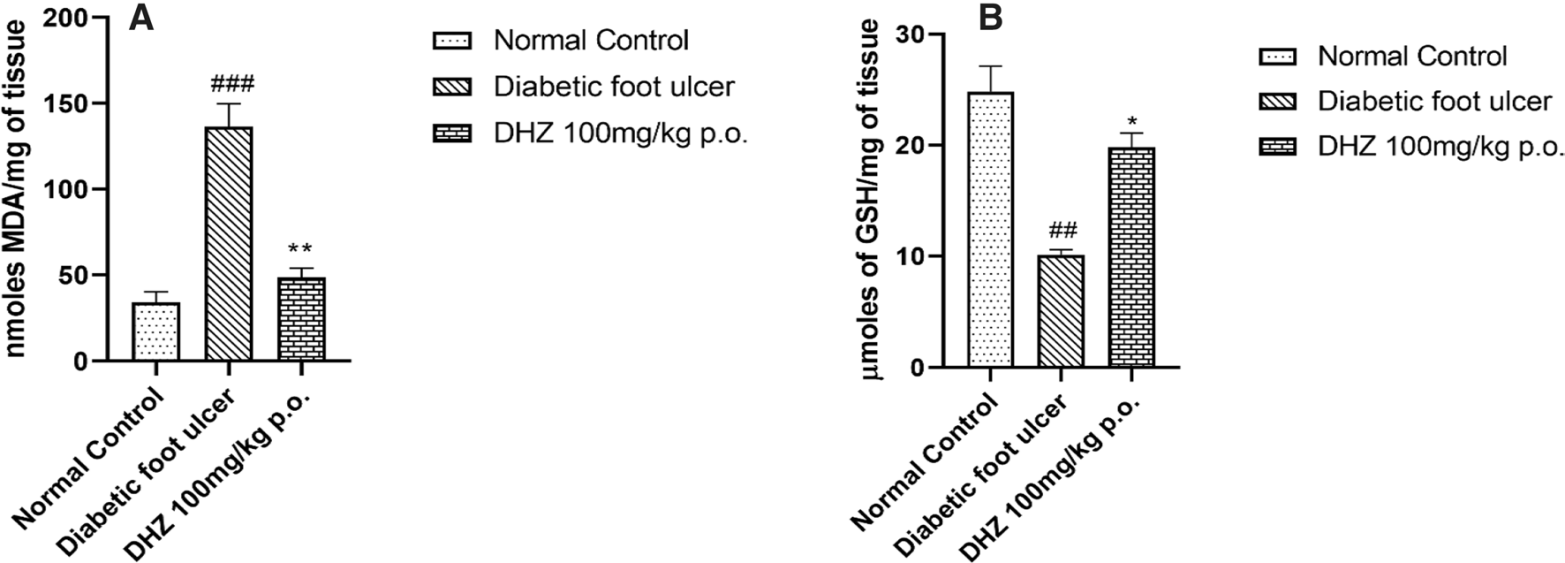
**A** Effect of DHZ on Malondialdehyde (MDA) levels on diabetic foot ulcer tissue **B** Effect of DHZ on GSH levels in diabetic foot ulcer tissue in type-II diabetic Wistar rats. Data represented as Mean ± SEM. **p < 0.001, *p < 0.01 when compared to HFD group and ^###^*p* < 0.0001, ^##^*p* < 0.001 compared to normal pellet fed diet. Analysis done by using one-way ANOVA with Tukey’s post-hoc test

Hyperglycemia tend to stimulate, the release of free radicals thus creating oxidative stress leading to insulin resistance (Semadi and Irawan 2017), formation of reactive oxygen species (ROS) and delaying the healing of diabetic foot. When present in low concentrations, ROS helps in the healing of wounds by activating cellular messengers like growth factors, cell migration & proliferation, synthesis of collagen and angiogenesis. (Oksigen Hiperbarik sebagai Terapi Adjuvan Kaki Diabetik Hendry Irawan 2016; Semadi and Irawan 2017) Whereas a higher concentration of ROS causes cellular damage, thus retarding the healing process (Dunnill et al. 2017). The higher formation of ROS is also due to lipid peroxidation which improves the regulation of tissue antioxidant enzyme activity (Ayala et al. 2014; Matsunami et al. 2010; Semadi and Irawan 2017; Tiwari et al. 2013). Among patients with diabetes, increase in lipid peroxidation due to chronic blood glucose levels and enhanced oxidative stress is observed. Due to enhanced oxidative stress on cellular components continuous cell damage occurs, thus leading to impaired healing(F et al. 2002; Schäfer and Werner 2008; Telorack et al. 2016a). We observed that animals treated with DHZ have shown significantly decreased levels of malondialdehyde (MDA) (48.63 ± 5.22) (*p* < 0.001) when compared with the disease group (136.4 ± 13.3) and in normal control MDA levels were found to be relatively low (34.23 ± 6.17) (Fig. 5A).

Detoxification of ROS decreases the oxidative stress generated on the cellular components which can be attained enzymatically or by using antioxidants. GSH (tripeptide Glutathione) is present in all cell types in high concentrations and acts as an antioxidant by detoxification of peroxides by acting as a co-factor to glutathione peroxidase (Schäfer and Werner 2008; Sies 1999; Telorack et al. 2016a). Many studies have suggested that GSH plays a very important role in wound healing by protecting the cell components under stress conditions (Adamson et al. 1996; Aktunc et al. 2010; Telorack et al. 2016a). Results showed decreased levels of GSH in the disease group (10.13 ± 0.44) when compared to the normal control group (24.83 ± 2.28) and treatment with DHZ was able to restore its levels (19.81 ± 1.27) significantly (*p* < 0.01) which indicate that DHZ can significantly elevate the levels of GSH which may be due to its antioxidant property (Fig. 5B).

Research studies suggest that antioxidants can diminish some of the vascular dysfunction caused due to hyperglycemia and reduce the oxidative stress developed on the cell by decreasing ROS formation. Studies done previously on the compound DHZ have shown antioxidant and anti-inflammatory activities (Fong et al. 2003; Fowler 2008; Hayun et al. 2018; Rao et al. 2011). According to our study results, DHZ showed a decrease in the TG, TC, LDL, FBG, MDA levels and an increase in HDL, GSH levels which indicate that DHZ has antioxidant activity and can decrease the chronic blood glucose levels, which can help the wound to heal at a faster rate.

### Effect of dehydrozingerone on TNF-α and IL-1β expression

The pro-inflammatory cytokines like TNF- α and IL-1β, IL-6, and IL-1α play a vital role in the process of wound healing by stimulating the migration and proliferation of fibroblasts, keratinocytes and production of extracellular matrix, chemotaxis of fibroblasts at the site of the wound and modulating the immune response (Agyare et al. 2018a; Werner and Grose 2003). These proinflammatory cytokines expression will be upregulated during the inflammatory phase of normal wound healing (Grellner et al. 2000). After the inflammatory phase, the expression of cytokines is downregulated. But in the case of diabetic and delayed wounds, the expression of pro-inflammatory cytokines continues to remain upregulated which enhances the inflammatory phase even longer than it occurs in normal healing. Thus delaying, the wound healing process. (Agyare et al. 2018a; Patel et al. 2016). We selected TNF**-** α & IL-1 as they are considered the masters of cytokines due to their efficiency to influence IL-6, Nitric oxide production (Gosselin and Rivest 2007; Thuraisingam et al. 2010).

**Fig. 6:**
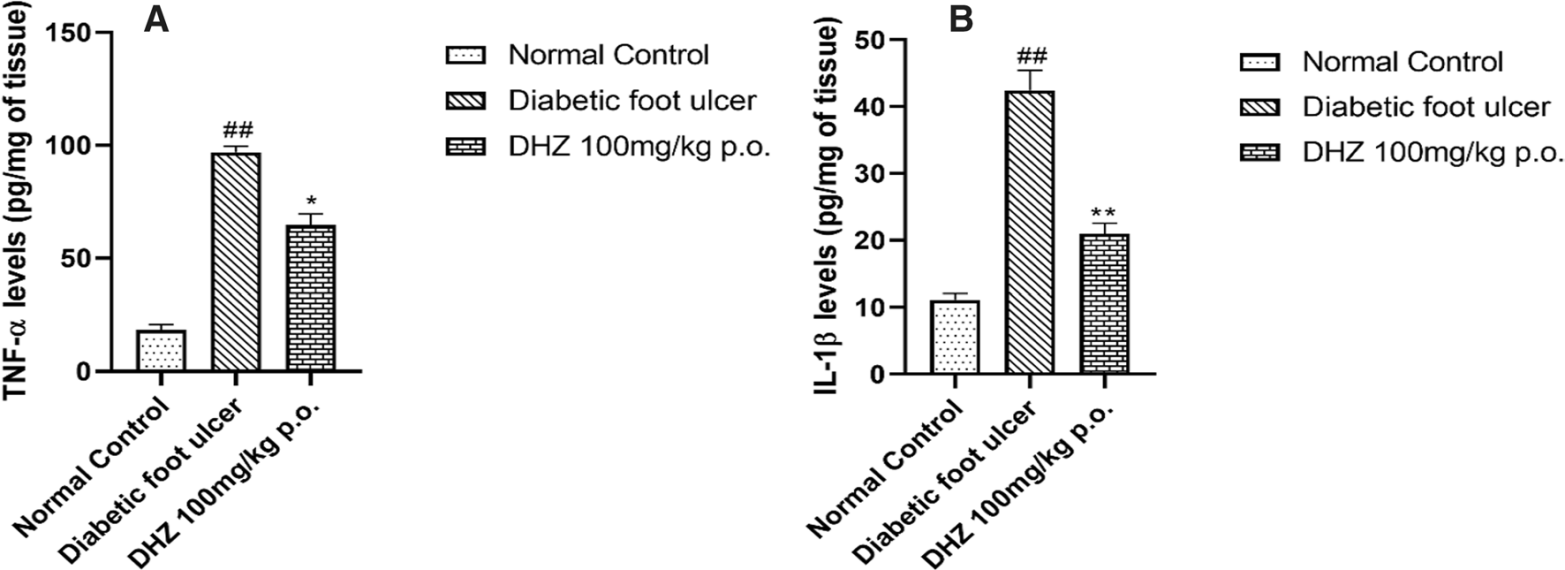
**A** Effect of DHZ on TNF-α levels on day 5 (post wounding) diabetic foot ulcer tissue **B** Effect of DHZ on IL-1β levels on day 5 (post wounding) in diabetic foot ulcer tissue. Data represented as Mean ± SEM. *p < 0.01, **p < 0.001 when compared to DFU group and ^##^*p* < 0.001 compared to normal control. Analysis done by using one-way ANOVA with Tukey’s post-hoc test

TNF- α is considered a key pro-inflammatory cytokine present in the initial phases of inflammatory responses occurring within the body. After the creation of the wound, TNF- α is expressed leading to an inflammatory response. Upregulation occurs within the first several hours of wound induction then reaches a peak followed by returning to its normal levels (Agyare et al. 2018a; Ritsu et al. 2017). Studies have shown that an increase in TNF- α along with  a decrease in IL-10 (it has anti-inflammatory properties) sustains the expression of chemokines leading to infiltration of leucocytes at wound site. Thus prolonging the inflammatory phase ultimately reducing the potential of the wound to heal (Gourishetti et al. 2020; Sen 2009; Xu et al. 2013). Our study showed that TNF- α levels on the 5th day of the wound were significantly high in the disease group when compared to normal and treated groups (Fig. 6A).

Macrophages release interleukin-1 at the site of injury or during any infection (Agyare et al. 2018a; Chamberlain et al. 2013) IL-1 activates the endothelial cells leading to increased synthesis of vascular adhesion molecules and combinedly infiltrates the wound site with monocytes and creates an inflammatory response (Cook-Mills et al. 2011; Lobmann et al. 2006). IL-1 also stimulates fibroblasts and thus controls the release of MMPs. MMPs degrade the extracellular matrix paving way for increased migration of monocytes and downregulating the inflammatory response as MMPs tend to degrade IL-1. In disease conditions, downregulation of inflammatory response does not occur, thus prolonging the inflammation phase (Agyare et al. 2018a; Lobmann et al. 2006). In this study, treatment with DHZ significantly decreased the expression of IL-1β when compared to the disease group (Fig. 6B).

### Immunoblotting

Healing of wound involves mainly the migration & proliferation of cells. It also includes serial activation of cytokines like TNF-α, interleukins, ERK1/2 (extracellular signal-regulated kinase), JNK (Jun N-terminal kinase) and p38 which helps in regulating cell migration and proliferation(Chen et al. 2013; He et al. 2012; Makino et al. 2010; Wu et al. 2016; Yue et al. 2011).

**Fig. 7:**
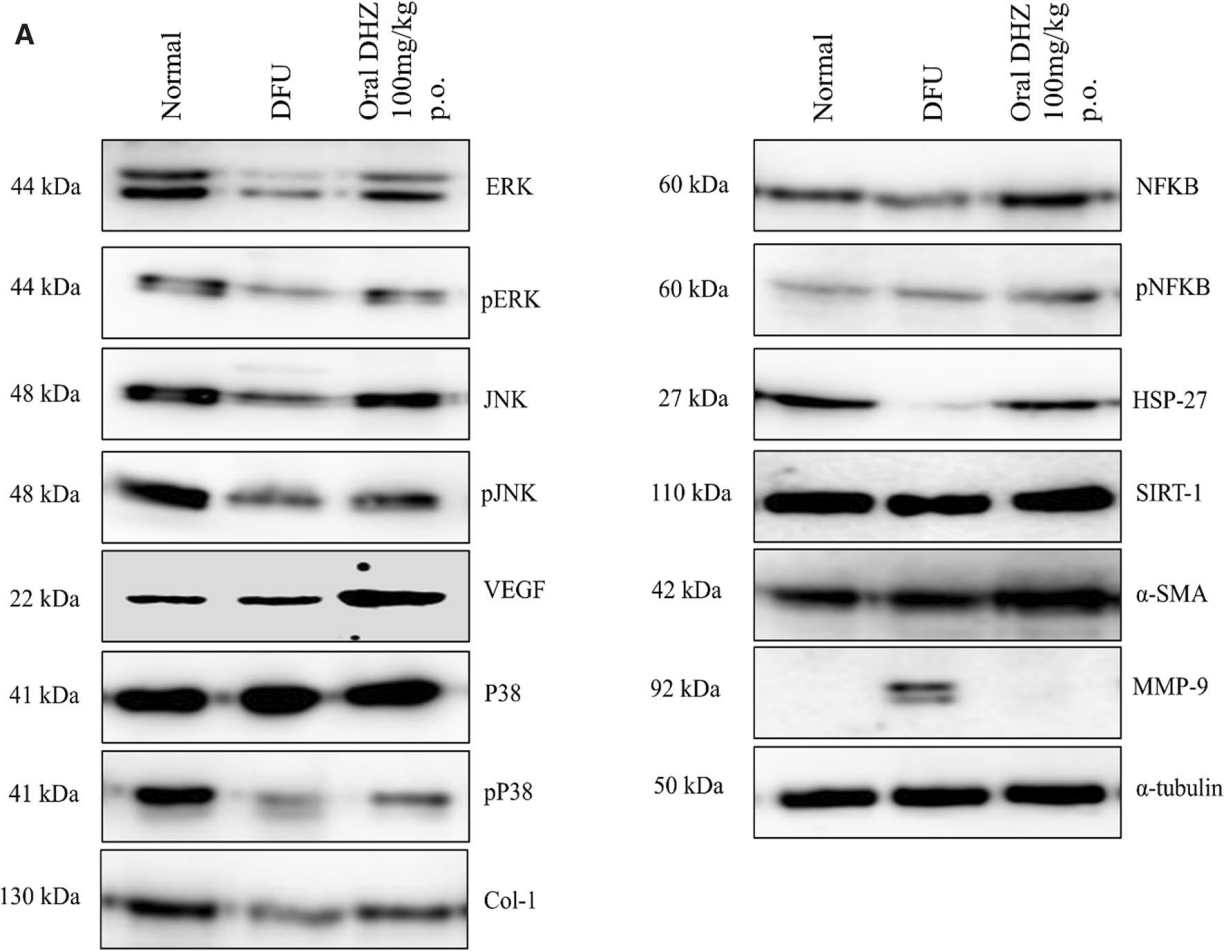

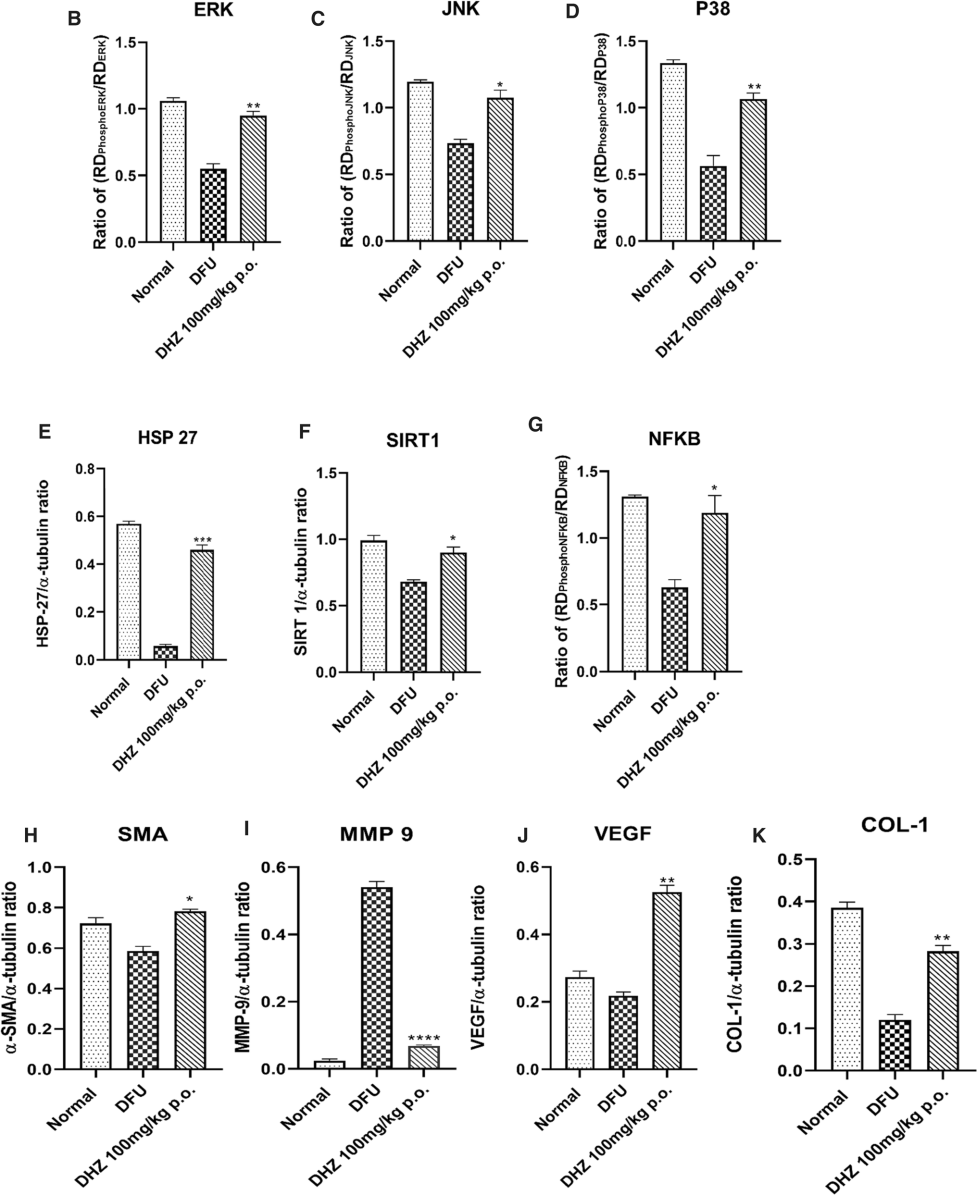
Effect of DHZ on MAPK, HSP-27, SIRT-1, NFkB, SMA-α, MMP-9, VEGF-B, COL-1 **A** Representative images of blots **B** p-ERK/ERK ratio, **C** p-JNK/JNK ratio, **D** p-P^38^/P^38^ ratio, **E** HSP-27/α-tubulin ratio **F** SIRT-1/α-tubulin ratio** G** p-NFkB/NFkB ratio **H** SMA- α /α-tubulin ratio **I** MMP-9/ α-tubulin ratio **J** VEGF-B/ α-tubulin ratio **K** COL-1. Data represented as Mean ± SEM. ***p < 0.0001, **p < 0.001, *p < 0.01 when compared to DFU group. Analysis done by using one-way ANOVA with Tukey’s post-hoc test.

These protein kinases can phosphorylate the cytoplasmic as well as nuclear targets. JNK/p38 gets activated due to stress, UV, proinflammatory cytokines, or heat shock and ERK1/2 gets activated because of mitogenic factors. Many studies suggested that these kinases are involved in the development, proliferation, migration, differentiation and survival of the cell (Sharma et al. 2003; Wu et al. 2016). Near the wound edge in the migrating epithelium, overexpression of p38 was noted whereas ERK1/2 activation was seen at the limbal area away from the wound (Nebreda and Porras 2000; Robinson and Cobb 1997; Sharma et al. 2003). Inhibition/downregulation of ERK1/2/JNK/p38 signalling pathway decreases the proliferation and migration of endothelial cells towards the wound edge resulting in impaired wound healing (Boulton et al. 1991; Ennis et al. 2005; Gazel et al. 2008; Harper et al. 2005; Kim et al. 2006; Wu et al. 2016). In the diabetic foot ulcer group, phosphorylation of ERK1/2/JNK/p38 was reduced when compared to the normal control group. DHZ significantly increased phosphorylation of ERK1/2/JNK/p38 when compared to disease control (Fig. 7B, C, D).

HSP-27 is one of the heat shock proteins which gets activated on exposure to stress. It recruits the dermal fibroblasts and thus maintains homeostasis at the wound area via MAPK signalling thus helping in the wound healing process. Due to chronic glucose levels in diabetic foot ulcers, the HSP-27 level gets downregulated thus impairing the process of healing (Brem and Tomic-Canic 2007; Gourishetti et al. 2020; Singh et al. 2015). Diabetic foot ulcer group showed reduced expression of HSP-27 when compared to normal control and DHZ group on day 5 (Fig. 7E).

SIRT-1 (Silent information regulator-1) [belongs to NAD + dependent histone deacetylase type III] is involved in the regulation of the inflammatory marker’s expression, re-epithelialization, granulation tissue formation, oxidative stress, proliferation and migration of keratinocytes (Debelec-Butuner et al. 2015; Li et al. 2019; Yang et al. 2015; Zhang et al. 2020a). DHZ significantly increased the expression of SIRT-1 compared to disease group. As SIRT-1 was a protein belonging to the upstream pathway of NF-kB, thus it inhibited NFkB phosphorylation. This indicates that DHZ increased the expression of SIRT-1 thereby decreasing the phosphorylation of NFkB (Fig. 7F, G). A study mentioned that upregulation of SIRT-1 and downregulation of NFkB inhibit the cytokines and MMPs expression (Zhang et al. 2020a). DHZ inhibited TNF-α & IL-1β levels as shown in Fig. 6A, B.

According to a research study, intriguingly both proteins show characteristics of incompatible crosstalk (Dvir-Ginzberg et al. 2011; Kauppinen et al. 2013). It means that NFkB is involved in pro-inflammatory response (Chalkiadaki and Guarente 2012) while SIRT-1 acts as an anti-inflammatory and are involved in cellular respiration (Kauppinen et al. 2013; Kornberg et al. 2010) which is essential for the wound to heal. NFkB gets activated due to ROS (oxidative stress) and thus shows an inflammatory response which plays a vital role in the initial phases of healing. In contrast, oxidative stress and inflammatory response can downregulate SIRT-1 activity (Caito et al. 2010; Kauppinen et al. 2013). This is what exactly happens in diabetic conditions, where SIRT-1 is downregulated and NFkB is upregulated due to oxidative stress created, stalling the wound in the inflammatory phase thus delaying healing process. DHZ restored the expression of SIRT-1 and inhibited NFkB phosphorylation thus reducing the inflammatory phase. (Cai et al. 2012; Kauppinen et al. 2013; Zhang et al. 2010). From western blotting, we demonstrated that DHZ controls the inflammatory phase by activating SIRT1 and inhibiting NFkB which transformed the wound's microenvironment thus promoting wound healing.

α-SMA (smooth muscle actin) is a protein present in the myofibroblasts (activated fibroblasts) and regulates extracellular matrix deposition, growth factors and matrix metalloproteinases (MMPs) and cytokines mainly involved in the healing process (Darby et al. 2014; Qiang et al. 2017; Werner et al. 2007) (Fig. 7H).

Literature showed that MMPs upregulation is one of the reasons for impaired wounds, mainly gelatinases/MMP-9 (Liu et al. 2018; Zhang et al. 2020a). MMPs degrade the extracellular matrix thus preventing the formation of scar. Overexpression of MMPs will completely degrade extracellular matrix thus preventing healing. Hence overexpression of MMPs harm the healing process. Finally, angiogenesis is very important for the wound to heal as the blood vessels carry the required nutrition and oxygen to the wound area helping in the process of healing. The disease group showed decreased expression of SMA, VEGF and overexpressed MMP-9 when compared to the normal group and treated group (Fig. 7I). DHZ significantly downregulated the MMP-9 expression and increased the expression of SMA and VEGF(Fig. 7J).(Ågren 1999; Mirastschijski et al. 2004; Pilcher et al. 1999a, 1999b, 1997; Sudbeck et al. 1997; Wu et al. 2016). Collagen formation is considered as one of the hallmarks of wound healing, Fig 7K showed the levels of Collagen-1 formation which was significant when compared to DFU group.

### Histopathology

Malfunction in the metabolism of collagen in diabetes conditions is thought to be one of the factors of impaired healing (Ahmad et al. 2017a). Impaired healing in diabetic rats is also due to high levels of metalloproteases, impairment in the formation of new blood vessels, dysfunction of epidermal cells and fibroblasts and decreased formation of granulation tissue (Ahmad et al. 2017a; Lodhi and Singhai 2013). Due to lipid peroxidation there will be an increase in the production of ROS and futile scavenging which even deteriorates healing conditions by modulating the proliferation and migration of fibroblasts (Ahmad et al. 2017a). Fibroblast, granulation tissue, angiogenesis and collagen formation are considered the hallmarks of wound healing (Gourishetti et al. 2020).

Histopathological studies were performed on days 5, 10 and 15 of DHZ treatment. Day 5 of DHZ treatment showed the presence of granulation tissue, inflammatory infiltrates whereas in the disease group oedematous areas and haemorrhagic areas were observed. Epithelium was discontinuous and 3–6 layers in thickness in the treated group was observed. On day 10 of treatment epithelium was formed properly with 4–5 layers in thickness and granulation tissue showing blood vessels, inflammatory cells and fibroblasts which were missing in the disease group. Day 10 of the disease group showed few fibroblasts and acute inflammatory cells when compared to the treatment group and normal group.

**Fig. 8:**
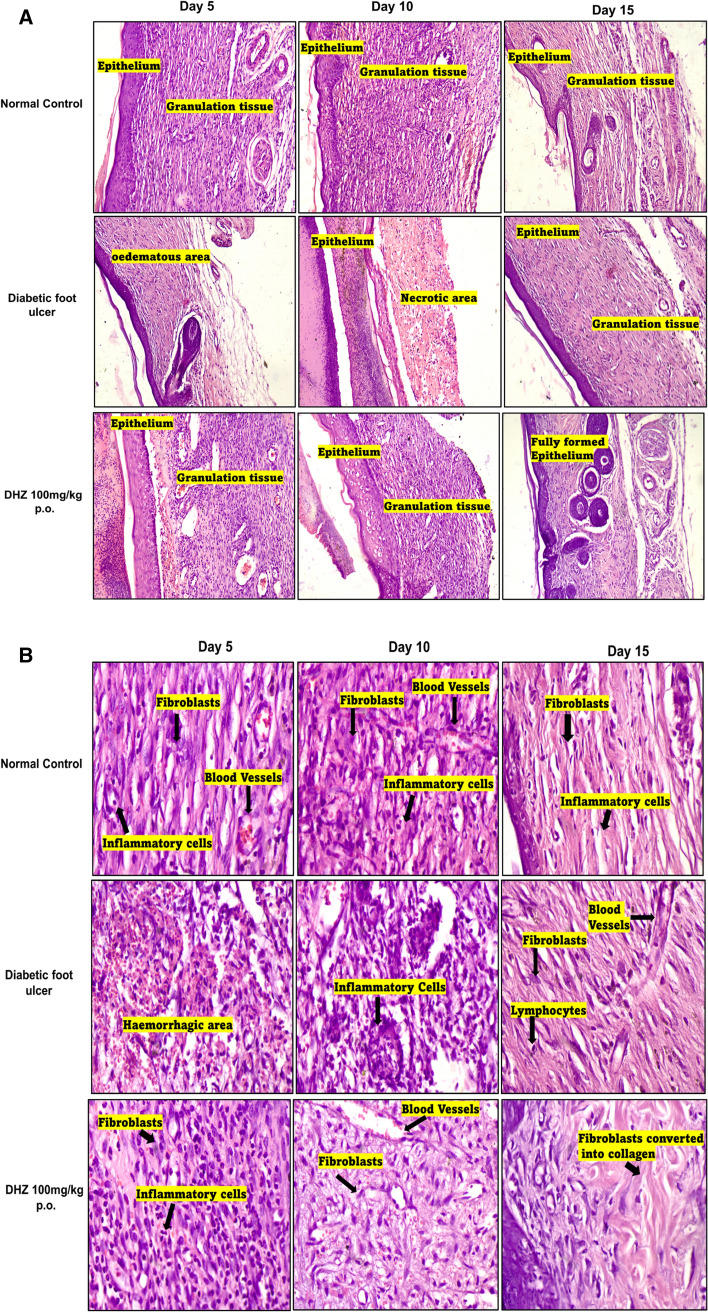
**A** Effect of DHZ on histopathology (H&E staining). Images captured at 100 × optical zoom **B** Effect of DHZ on histopathology (H&E staining). Images captured at 400 × optical zoom

Day 15 of drug treatment showed formation of complete epithelium which was stratified squamous keratinized consisting of 2 to 3 layers. Most of the wound area transformed into normal skin and dermis showed presence of normal collagen bundles which were not observed in the disease group. In the disease group on Day 15, the granulation tissue showed formation of new blood vessels and fibroblasts with few lymphocytes which were seen on day 10 itself in the treatment group (Fig. 8). The degree of healing was measured by grading histological parameters in which it was observed that grading was good in the treatment group when compared to the disease group (Table 1).

**Table 1:** Grading done for various features as follows:  + slight; +  + moderate; +  +  + extensive; − absence

Groups	S.No	Histopathology Parameter	Day 5	Day 10	Day 15
Normal Control	1	Scab	−	−	−
	2	Epidermal regeneration	+++	++	+++
	3	Granulation tissue	++	++	+++
	4	Inflammatory cell infiltration	+	+	+
	5	Angiogenesis	+	++	+++
	6	Proliferation of fibroblasts	++	++	+++
	7	Collagen deposit	++	++	+++
Diabetic foot ulcer	1	Scab	+++	++	−
	2	Epidermal regeneration	+	+	+++
	3	Granulation tissue	−	+	++
	4	Inflammatory cell infiltration	−	++	+
	5	Angiogenesis	−	+	++
	6	Proliferation of fibroblasts	−	+	++
	7	Collagen deposit	−	+	+
DHZ 100mg/kg p.o.	1	Scab	++	+	−
	2	Epidermal regeneration	++	++	+ + +
	3	Granulation tissue	++	+	+++
	4	Inflammatory cell infiltration	++	+	+
	5	Angiogenesis	+	++	+ ++
	6	Proliferation of fibroblasts	+	++	+ ++
	7	Collagen deposit	+	++	+++

**Fig. 9:**
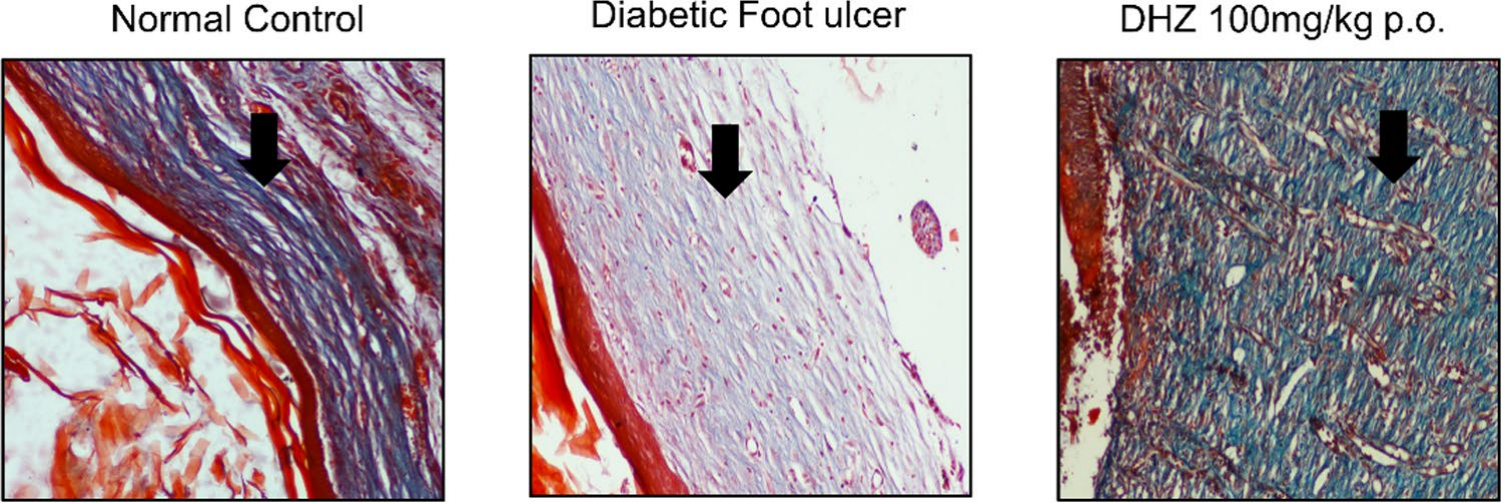
Effect of DHZ on Collagen deposition (Masson trichrome staining). Images captured at 400 × optical zoom

Masson trichrome staining is done to estimate the amount of collagen deposition at the wound site. We performed Masson trichrome staining on day 15 in all the three groups, where the treatment and normal control group showed more collagen formation when compared to the disease group (Fig. 9).

The epidermal regeneration, deposition of collagen, angiogenesis and ECM production are the hallmarks of wound healing in humans. Our study results have shown significant improvement in the above mentioned parameters. Thus we suggest that dehydrozingerone can be experimented further in clinical trials involving diabetic wounds.

## Conclusion

Dehydrozingerone accelerates the wound healing in diabetic foot ulcers.

## Data Availability

Not Applicable.
